# Site-2 protease Sll0528 interacts with RbcR to regulate carbon/nitrogen homeostasis in the cyanobacterium *Synechocystis* sp. PCC 6803

**DOI:** 10.3389/fmicb.2025.1556583

**Published:** 2025-04-09

**Authors:** Shiqi Lin, Taotao Zheng, Yongyi Mo, Ge Zhang, Gu Chen

**Affiliations:** School of Food Sciences and Engineering, South China University of Technology, Guangzhou, China

**Keywords:** cyanobacteria, proximity labeling, site-2 protease, Sll0528, RbcR, *Synechocystis*

## Abstract

Cyanobacteria play pivotal roles in global biogeochemical cycles through oxygenic photosynthesis. To maintain cellular homeostasis, these organisms utilize sophisticated acclimation mechanisms to adapt to environmental fluctuations, particularly concerning nitrogen availability. While nitrogen deprivation induces dormancy, excess ammonium can have toxic effects on cyanobacteria and other photosynthetic organisms—a phenomenon for which the acclimation mechanisms remain poorly understood. Through the physiological characterization of knockout and overexpression mutants in *Synechocystis* sp. PCC 6803, we identified the site-2 protease Sll0528 as a critical regulator of ammonium stress acclimation. TurboID-based proximity labeling, coupled with quantitative proteomics, revealed a robust set of putative Sll0528-interacting proteins, some of which were subsequently validated through bacterial two-hybrid assays and transcriptomic profiling. Notably, we confirmed the physical interaction between Sll0528 and RbcR, a low-carbon-responsive transcriptional regulator. Transcriptomic analysis showed that the knockout of *sll0528* led to a significant downregulation of the RbcR regulon, including the ribulose-1,5-bisphosphate carboxylase/oxygenase (RuBisCO) operon *rbcLXS*. Further analysis suggests that this downregulation might result from improper post-transcriptional regulation of RbcR, which depends on its interaction with Sll0528. Our findings reveal novel regulatory crosstalk between a cyanobacterial S2P protease and the carbon-responsive transcriptional machinery, providing new mechanistic insights into the control of cyanobacterial carbon-nitrogen homeostasis during nitrogen fluctuations. This study offers insights into the functional characterization of other S2P proteases in photosynthetic organisms and may facilitate the cyanobacteria-based bioremediation of ammonium-rich wastewater.

## Introduction

Cyanobacteria are ancient and important photosynthetic organisms in the global ecosystem. They serve as useful models for studying metabolic and physiological processes conserved across photosynthetic organisms and hold potential as renewable resources for producing valuable chemicals (Bolay et al., 2024). Characterizing cyanobacterial physiology and metabolism is crucial for understanding their environmental roles and unearthing their potential for biotechnology applications (Esteves-Ferreira et al., 2018; Cassier-Chauvat et al., 2021; Sharma et al., 2022). Thus, investigations into their acclimation mechanisms to fluctuating nutrient levels, particularly with regard to the two most abundant elements—carbon and nitrogen—provide scientific insights and opportunities for metabolic engineering in cyanobacteria.

Studies focusing primarily on nitrogen depletion have revealed a coordinated control of carbon and nitrogen homeostasis by a sophisticated network, in which the widespread signaling protein PII acts as a major regulatory hub (Forchhammer et al., 2022). PII, together with the global nitrogen transcriptional regulator NtcA and their interacting proteins, such as PipX and PirC, monitor the intracellular concentrations of metabolites such as 2-oxoglutarate, cAMP, and ATP/ADP, switching their protein–protein interactions with the downstream metabolic enzymes, transporters, and regulators to maintain carbon and nitrogen homeostasis (Esteves-Ferreira et al., 2018; Zhang et al., 2018; Forchhammer and Schwarz, 2019; Watzer et al., 2019; Forchhammer and Selim, 2020; Selim et al., 2020; Orthwein et al., 2021; Forchhammer et al., 2022; Diez et al., 2023). However, limited research has been conducted on cyanobacterial acclimation to surplus nitrogen, especially at high concentrations of ammonium. Ammonium is a major nutrient, but high concentrations of it are toxic to many organisms, particularly plants and oxygenic photosynthetic microorganisms (Xie et al., 2023). Ammonium has been found to trigger photodamage to photosystem II (PSII), with the oxygen-evolving complex of PSII suggested as the direct target of ammonium toxicity (Drath et al., 2008). Efficient PSII repair by D1 proteins encoded by three genes, *psbA1*, *psbA2,* and *psbA3*, is crucial for ammonium tolerance in *Synechocystis* PCC 6803 (Dai et al., 2014).

Overuse of NO_3_^−^ and NH_4_^+-^based fertilizers and improper discharge of animal waste and industrial effluent have resulted in global nitrogen pollution as a serious worldwide issue of public and economic concern, such as the eutrophication of water reservoirs, soil contamination, and atmospheric pollution (Erisman et al., 2013; Erisman, 2021; Varjani et al., 2021; Gu et al., 2023). As an economical and sustainable approach to mitigating nitrogen pollution, cyanobacteria can utilize high-nitrogen-containing wastewater to produce potentially valuable biomass synergistically (Bhatia et al., 2021; Priyadharshini et al., 2021; Sharma et al., 2022). Nevertheless, the ammonium acclimation mechanism in cyanobacteria remains elusive. Notably, we recently identified cyanobacterium *Synechocystis* sp. PCC 6803 (hereafter referred to as *Synechocystis* 6,803) site-2 protease (S2P) Slr1821 as an essential regulator that coordinate carbon/nitrogen homeostasis during ammonium stress acclimation (Lin et al., 2023). However, its substrate or direct interacting protein remains unknown.

Site-2 protease (S2P) is named after the human S2P, which performs intramembrane proteolysis of the precursor of transcription factor SREBP-2 after site-1 protease (S1P) to regulate lipid metabolism (Sakai et al., 1996). S2Ps are prevalent across all three domains of life. Belonging to the group of intramembrane proteases, S2Ps cleave their substrates to regulate signal transduction and maintain proteostasis (Brown et al., 2000; Kuehnle et al., 2019). For example, the *Escherichia coli* S2P RseP cleaves the anti-sigma factor to release the sigma factor in response to extracytoplasmic stress; RseP also degrades small membrane proteins and suppresses the cytotoxicity of the intrinsic toxin HokB (Imaizumi et al., 2022; Yokoyama et al., 2023). S2P homologs are widely distributed in the phylum cyanobacteria, suggesting their essential roles (Chen and Zhang, 2010). *Synechocystis* 6,803 has four S2Ps; other than the aforementioned Slr1821, which is involved in ammonium stress acclimation, Slr0643 and Sll0528 were found essential for acclimation to acid and salt stress, respectively (Zhang et al., 2012; Lei et al., 2014). However, the direct downstream targets of these S2Ps, such as their substrates or interacting proteins, remain poorly understood, hindering further characterization of cyanobacterial S2Ps.

Coimmunoprecipitation (Co-IP) has been successfully applied to identify a wide range of interacting proteins. For example, Co-IP revealed that the ammonium, nitrate, and urea transporters interact directly with PII (Watzer et al., 2019), while Co-IP further identified PirC as a novel PII interactor, which switched its interaction with PII to bind and inhibit phosphoglycerate mutase upon nitrogen starvation, thus controlling carbon storage metabolism (Orthwein et al., 2021). However, S2Ps contain multiple transmembrane domains, and harsh conditions are required to disrupt membranes and enrich such integral membrane proteins, thus making Co-IP unsuitable for identifying their interacting proteins since most protein–protein interactions cannot be maintained under severe conditions. Alternatively, the proximity labeling approach could address this issue and reveal the transient interacting proteins (Qin et al., 2021; Milione et al., 2024). In proximity labeling, a promiscuous enzyme is fused in-frame with the bait protein to co-express and tag the endogenous interaction partners of the bait *in vivo*. Tagged molecules that interact with the bait are then enriched and identified by mass spectrometry for proteins or sequencing for nucleic acids. For example, peroxidase (APEX) and biotin ligase (TurboID, BioID) have been used to study protein–protein interaction in various animal and plant living systems (Lam et al., 2015; Kim et al., 2016; Branon et al., 2018; Zhang et al., 2019; Bosch et al., 2021; Zhang et al., 2022). In the presence of ATP, TurboID catalyzes the conversion of biotin to biotinoyl-5’-AMP, subsequently binding to lysine residues in prey proteins near the bait (Branon et al., 2018).

In this study, we applied TurboID to explore the directly interacting proteins of cyanobacterial S2P. In addition to its critical role in salt stress response, Sll0528 of *Synechocystis* 6,803 was also found to play an essential role in acclimation to high ammonium stress. Therefore, this study aimed to reveal the interacting proteins of Sll0528 and provide insights into carbon/nitrogen homeostasis control during fluctuations in nitrogen levels. Although TurboID has been demonstrated in various animal and plant models, this is the first application of TurboID-based proximity labeling in cyanobacteria. It provides new insights into the functional characterization of photosynthetic S2P and may facilitate the bioremediation of ammonium-rich wastewater using cyanobacteria in the future.

## Materials and methods

### Strains and cultivation

*Synechocystis* sp. PCC 6803 GT-G strain, derived from ATCC 27184, was used as the wild type (WT) (Ding et al., 2015). Cells were cultivated in BG11 medium (Rippka et al., 1979) at pH 7.5, buffered with 20 mM HEPES, at 29°C under ambient CO_2_ and constant illumination (30 μE·m^−2^·s^−1^). Mutant strains were cultivated in the presence of 50 μg mL^−1^ kanamycin or 80 μg mL^−1^ chloramphenicol. For ammonium stress experiments, the cells were cultured in BG11 medium supplemented with various concentrations of NH_4_Cl, maintaining pH 7.5 and buffered with 20 mM HEPES.

### Generation of recombinant *Synechocystis* strain

The *sll0528* gene knockout and overexpression strains were generated through natural transformation and homologous recombination (Lei et al., 2014). The *psbA2* promoter drives the constitutive expression of the *sll0528* gene in the overexpression strain, *OEsll0528*.

The plasmids p0528 and pTBYPC were constructed to generate recombinant *Synechocystis* strains P0528T and TBYPC (Supplementary Figure S1 and Supplementary Table S1). For the construction of p0528, the *sll0528* gene and its upstream and downstream regions were PCR amplified from wild-type (WT) *Synechocystis* DNA using specific primers (Supplementary Table S2). The TurboID gene was generated through DNA synthesis according to the reported sequence (Branon et al., 2018), and the kanamycin resistance gene (Kana) was obtained from pET-30b. These fragments were sequentially inserted into pUC118 to create p0528, which was subsequently introduced into the WT strain via homologous recombination. As a control, the TurboID gene was inserted into the self-replicating vector pJA2C to create the plasmid pTBYPC, which was subsequently introduced into the WT strain using electroporation. After screening with specific antibiotics, the recombinant strains were confirmed through PCR and sequencing.

### TurboID-based proximity labeling and enrichment of biotinylated proteins

Proximity labeling using TurboID was mainly conducted according to a previous report (Cho et al., 2020), with some modifications. Labeling was performed in a BG11 medium containing 1 mM ATP, 5 mM MgCl_2_, and varying concentrations of biotin for the desired time in a light incubator. After labeling, the supernatant was removed by centrifugation (8,000 rpm for 6 min), and the cell pellets were gently washed five times with ice-cold PBS (phosphate-buffered saline, pH 7.4). The cell pellets were resuspended in 1 mL of RIPA (Radio Immunoprecipitation Assay) lysis buffer supplemented with 1 x protease inhibitor cocktail, 1 mM PMSF (Phenylmethylsulfonyl fluoride, a protease inhibitor), 5 mg mL^−1^ lysozyme, 2 μg mL^−1^ endonuclease Benzonase, and 0.01% Triton X-100. The cell pellets were lysed using a Tissuelyser (Jingxin, Shanghai, China) with 500 μL ceramic beads (70 Hz, 300 s, repeated 7–8 times) and repeated freezing in liquid nitrogen. The lysates were then incubated on ice for ≥10 min. The cell lysates were clarified by centrifugation at 12,000 rpm at 4°C for 10 min, and the clarified lysates were transferred to fresh tubes. Protein concentration was determined using the BCA protein assay as previously described (Cortes-Rios et al., 2020). The protein solution was filtered through a 0.22 μm filter and stored at −80°C.

Biotinylated proteins were enriched using a Streptavidin Beads 6FF gravity column according to the manufacturer’s instructions with some modifications. The gravity column was first equilibrated with RIPA lysis buffer, and the protein samples were loaded onto the column and incubated overnight at 4°C with rotation for 1 h overnight. The column was washed with RIPA lysis buffer, and the enriched proteins were eluted with 3 × protein loading buffer supplemented with 2 mM biotin and 20 mM DTT (Dithiothreitol) at the desired temperature for 10 min. The input, flow-through, and eluate samples were analyzed by SDS-PAGE and Western blot to confirm the depletion of biotinylated proteins from the flow-through and their enrichment in the eluate. The eluate was lyophilized and stored at −80°C.

### Western blot analysis

Protein samples in 1 x protein loading buffer were boiled at 95°C for 10 min and separated electrophoretically on a 12% SDS-PAGE gel. The proteins were then transferred to a PVDF membrane. The membrane was stained with Ponceau S solution to confirm transfer quality and protein loading, destained with deionized water, and washed three times for 10 min each with TBST (0.05 M Tris, 0.15 M NaCl, 0.05% Tween 20). It was blocked overnight at 4°C with 5% (w/v) nonfat milk powder in TBST while constantly rotating. After blocking, the membrane was washed three times for 10 min each with TBST to remove any residual nonfat milk, then incubated with Streptavidin-HRP in TBST containing 3% BAS for 30 min at room temperature or overnight at 4°C. The membrane was washed three times for 10 min each with TBST, and the signal was developed using ECL western blot substrate for the necessary duration and imaged.

### 4D label-free quantitative proteomic analysis

The protein samples were dissolved in triethylammonium bicarbonate buffer (TEAB) and digested overnight with trypsin at 37°C. After trypsin digestion, the peptides were vacuum-dried, reconstituted in trifluoroacetic acid, desalted, and vacuum-dried again. The peptides were analyzed using liquid chromatography–tandem mass spectrometry (LC–MS/MS) with the NanoElute UPLC system (Bruker, Germany), incorporating a capillary ion source and were analyzed with the timsTOF Pro2 mass spectrometer (Bruker, Germany). The electrospray voltage was set to 1.5 kV. Following nanoscale HPLC separation, the samples were analyzed by data-dependent acquisition (DDA) mass spectrometry, utilizing high-resolution TOF to detect and analyze peptide parent ions and their secondary fragments. The secondary MS scanning range was set to 100–1700 m/z. Data were acquired using the parallel accumulation serial fragmentation (PASEF) acquisition mode on the timsTOF Pro2, with the second MS stage recorded using the 10 PASEF mode.

The raw MS/MS spectral data were searched against the *Synechocystis* 6,803 protein database (UniProt version 2022.09) using MaxQuant (v.2.6.1.0). The subsequent quantitative analysis included proteins with at least one unique peptide matched. The expression fold change between different groups and the statistical significance, based on the FDR and *p*-value, were calculated using ANOVA followed by Student’s *t*-test. An enrichment analysis of proteins annotated in the eggNOG database was conducted to identify significantly enriched categories based on the corrected *p*-value <0.05.

### Bacterial two-hybrid assay

The bacterial two-hybrid vectors pUT18 and pKT25, containing the genes for either the T18 or T25 subunit of adenylate cyclase CyaA (Karimova et al., 2001), were used to construct plasmids. Genomic DNA from *Synechocystis* 6,803 served as the template. The coding regions for Sll0528 and the selected putative interacting proteins were amplified using high-fidelity DNA polymerase and inserted into pUT18 and pKT25, respectively (Supplementary Figure S2). Both C-terminal and N-terminal fusions of the tag to the gene of interest were prepared. The plasmids and primers used in this study are listed in Supplementary Tables S1, S2.

*Escherichia coli* BTH101 cells were co-transformed with recombinant plasmids pUT18 and pKT25 (Supplementary Figure S2). Co-transformants were screened on LB plates supplemented with 100 μg mL^−1^ ampicillin and 50 μg mL^−1^ kanamycin and cultivated for 2 days at 30°C. Six clones from each plate were picked and inoculated into 5 mL of LB medium containing antibiotics to minimize heterogeneity. After overnight cultivation, the cells were diluted 1:100 in 5 mL of fresh LB medium containing antibiotics and grown until the OD_730_ reached an OD of 0.8. Three μL of each culture were plated on X-Gal reporter plates containing antibiotics, 100 μg mL^−1^ IPTG, and 100 μg mL^−1^ X-Gal and cultivated for 2 days at 30°C.

### RNA extraction and quantitative RT-PCR

Total RNA was extracted as previously described (Lin et al., 2023). Residual DNA was removed using DNase and verified by PCR amplification without reverse transcription. Quantitative RT-PCR was performed using a one-step SYBR Green I kit (TAKARA Biotech, Dalian, China) on ABI7500 (Life Technologies, Grand Island, NY, USA) as previously described (Lin et al., 2023). The *rnpB* gene, which encodes the RNA subunit of ribonuclease P, served as an internal control. The primers used are listed in Supplementary Table S2. At least three independent biological replicates for each sample, and three technical replicates per biological replicate were analyzed. The relative expression of genes was calculated using the 2^−ΔΔCt^ method.

### Transcriptomic analysis

Total RNA was extracted using TRIzol® Reagent (Invitrogen, Carlsbad, CA, USA) according to the manufacturer’s instructions, and genomic DNA was removed using DNase I (Takara, Osaka, Japan). RNA quality was assessed with the 2,100 Bioanalyzer (Agilent, Santa Clara, CA, USA) and quantified using the ND-2000 (NanoDrop Technologies, Waltham, MA, USA). High-quality RNA samples were used to construct sequencing libraries with the TruSeq™ RNA sample preparation kit (Illumina, San Diego, CA, USA). Ribosomal RNA (rRNA) was depleted using the Ribo-Zero Magnetic kit (Epicenter), and total mRNAs were fragmented into short segments of approximately 200 nt with TransNGS® RNA Fragmentation Buffer (TransNGS, Beijing, China). Double-stranded cDNA was synthesized with random hexamer primers using a SuperScript double-stranded cDNA synthesis kit (Invitrogen, Carlsbad, CA, USA). Libraries were size-selected for cDNA target fragments of approximately 200 bp and subjected to PCR amplification using Phusion DNA polymerase (Finnzymes, Espoo, Finland) for 15 cycles. After quantification, the paired-end RNA sequencing libraries were sequenced with Illumina HiSeq×TEN at a read length of 2 × 150 bp. The Illumina GA Pipeline (version 1.6) processed original images into sequences, performed base-calling, and calculated quality values to obtain 150 bp paired-end reads. After removing low-quality sequences, clean reads of each sample were mapped to the reference genome using Bowtie2.^1^ The expression level of each gene was computed in FPKM (fragments per kilobase of exon per million mapped reads) utilizing RSEM.^2^ Differential expression analysis among groups was conducted based on FPKM using edgeR.^3^

### Statistical analyses

The experiments were conducted in at least triplicate. The data presented are the mean values along with standard deviations. To assess statistical significance, a one-way analysis of variance (ANOVA) followed by an LSD test at a significance level of *p* < 0.05 was used.

## Results

### Sll0528 plays an essential role in the acclimation to high ammonium stress

**Figure 1:**
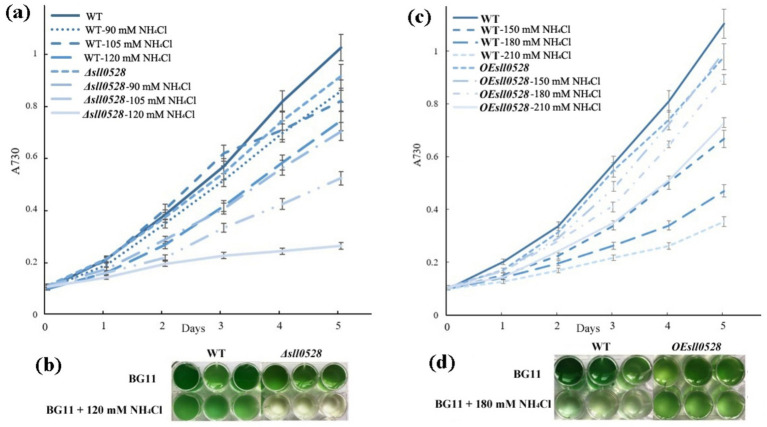
Knockout or overexpression of the *sll0528* gene decreased or increased ammonium stress tolerance, respectively. **(a)** The growth curve indicated that the *sll0528* knockout mutant (*Δsll0528*) was more sensitive to ammonium stress compared with the wild type (WT). **(b)** The phenotype of WT and *Δsll0528* with or without 120 mM NH_4_Cl on the fifth day as in **(a)**. **(c)** The growth curve indicated that the *sll0528* overexpression strain (*OEsll0528*) was more tolerant to ammonium stress compared with WT. **(d)** The phenotype of WT and *OEsll0528* with or without 180 mM NH_4_Cl on the fifth day as in **(c)**.

Although ammonium is the preferred nitrogen source for *Synechocystis* 6,803, it becomes toxic at high concentrations. High concentrations of ammonium (90 ~ 210 mM NH_4_Cl, as investigated in this study) inhibited the growth of *Synechocystis* 6,803, and the inhibition became more and more severe with increasing concentration (Figure 1). Interestingly, the deletion of the site-2 protease gene *sll0528* impaired ammonium acclimation, as the *Δsll0528 strain* could not survive moderate ammonium stress, which only slightly affected the growth of the WT (Figures 1a,b). In contrast, overexpression of *sll0528* substantially improved ammonium tolerance. The *OEsll0528 strain* proliferated much more rapidly than WT under high ammonium stress (150–210 mM NH_4_Cl) (Figures 1c,d). These results suggest that Sll0528 plays a critical role in ammonium acclimation.

### Construction of the Sll0528-TurboID line to reveal the Sll0528 interacting protein

**Figure 2:**
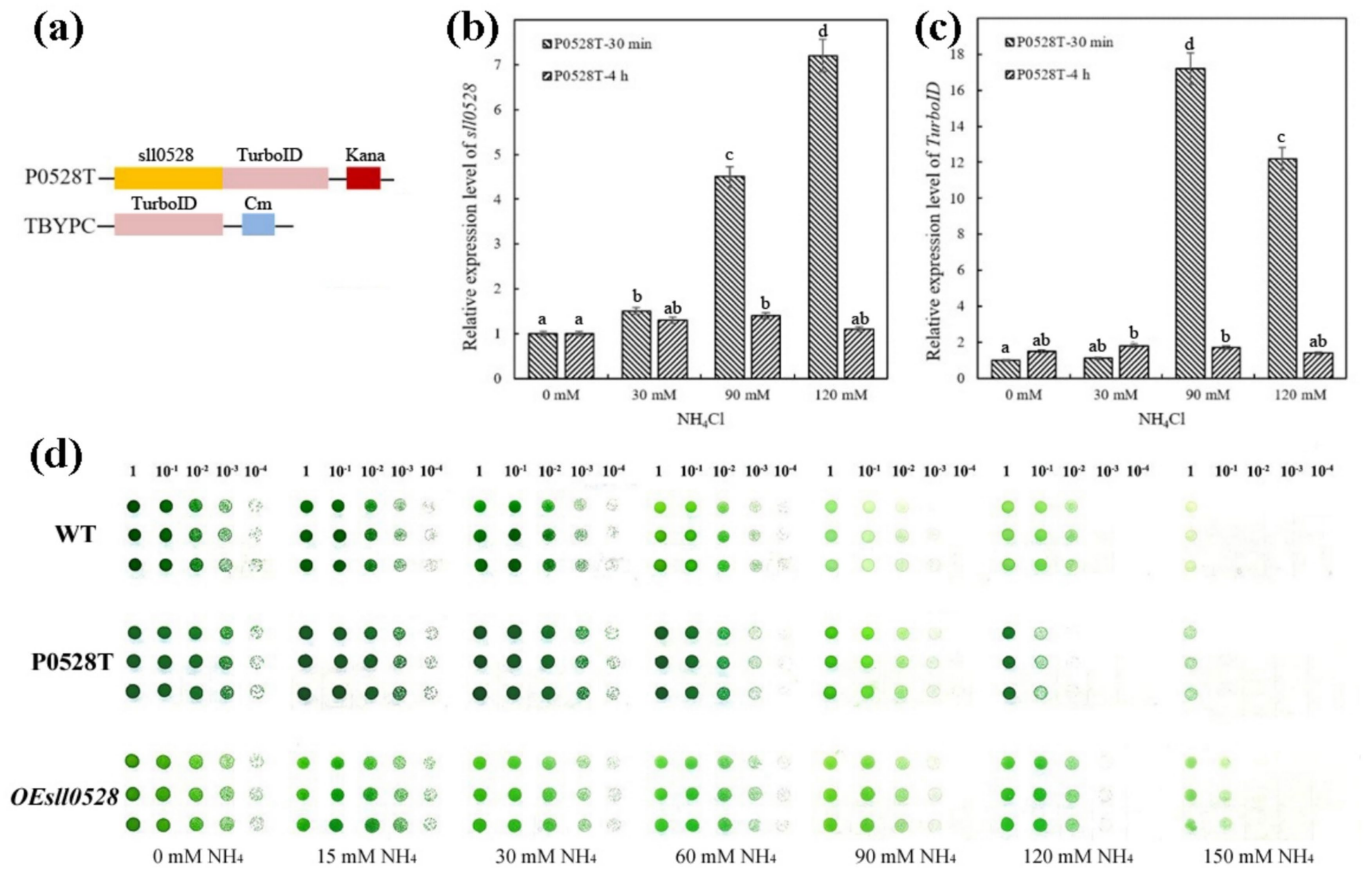
Ammonium-induced synchronous expression of *sll0528* and TurboID gene in P0528T. **(a)** Construction of the P0528T line and the control line TBYPC. The TurboID gene was fused in-frame with the *sll0528* gene in P0528T, while it was expressed by itself under the *psbA2* promoter in TBYPC. **(b)** and **(c)** Quantitative RT-PCR revealed the synchronous expression of *sll0528* **(b)** and TurboID **(c)** in P0528T induced by 90 mM and 120 mM NH_4_Cl. The columns with different letters were significantly different. **(d)** Ammonium sensitivity of the various *Synechocystis* strains was determined by the drop plate method with triple replicates of each strain. Cultures were adjusted to an OD_730_ of 1.0 and diluted tenfold in series before being dropped onto BG-11 agar plates containing different concentrations of extra ammonia (15–150 mM).

To investigate the underlying mechanism of Sll0528 during ammonium acclimation, the gene encoding the modified biotin ligase TurboID was fused in-frame downstream of the *sll0528* gene under its native promoter and transformed into WT to construct the P0528T line (Figure 2a and Supplementary Figure S1). Consistent with the ammonium-induced *expression* of sll0528 in WT, the P0528T line showed that 90 mM and 120 mM NH_4_Cl rapidly and dramatically induced sll0528 expression at 30 min, while the induction decreased significantly at 4 h (Figure 2b). Similarly, the ammonium-induced upregulation of TurboID in the P0528T line was prominent at 30 min following treatment with 90 mM and 120 mM NH_4_Cl, demonstrating the successful construction of the P0528T line (Figure 2c). Based on these data, a 30-min treatment with 90 mM NH_4_Cl was chosen for further analysis of the Sll0528 proximate proteins. Additionally, the spot assay revealed that the P0528T line exhibited ammonium tolerance comparable to that of WT (Figure 2d), suggesting that the functionality of the Sll0528 protein was not compromised by its fusion with TurboID. To eliminate randomly biotinylated proteins from the Sll0528 proximate proteins, the TBYPC line was constructed to constitutively express a soluble TurboID, serving as the background control line (Figure 2a and Supplementary Figure S1).

### Optimization of TurboID-based proximity labeling and enrichment of biotinylated proteins

**Figure 3:**
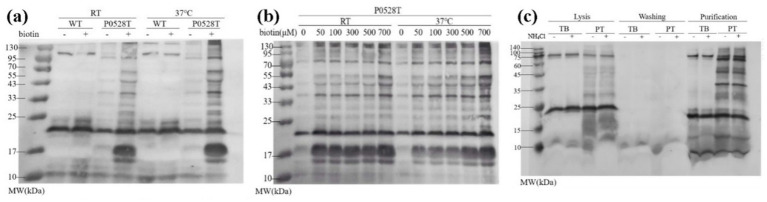
Optimization of biotin labeling and enrichment of biotinylated proteins. Biotinylated proteins were revealed by the antibody streptavidin-HRP in a western blot. **(a)** Comparison of biotin labeling effect under room temperature (RT) or 37°C in wild type (WT) and P0528T. **(b)** Comparison of labeling effect with different concentrations of biotin under RT or 37°C in P0528T. **(c)** Enrichment of biotinylated proteins from TBYPC and P0528T with (+) or without (−) 90 mM NH_4_Cl. Lysis: total proteins before streptavidin column enrichment; washing: unconjugated protein through the streptavidin column; purification: purified proteins eluted from the streptavidin column. TB: proteins extracted from the TBYPC line; PT: proteins extracted from the P0528T line. At least three independent batches of samples were prepared for each group.

The optimization of TurboID-based proximity labeling initially involved examining the effects of labeling temperature, biotin concentration, and labeling time. SDS-PAGE analysis of total protein indicated that there was no significant difference between WT and P0528T before or after biotin labeling. However, their biotinylated protein patterns were remarkably different, as revealed by a western blot using streptavidin-HRP as an antibody against biotinylated proteins (Supplementary Figures S3a,b). In the WT sample, the endogenous biotinylated protein patterns were similar before and after the biotin label, whereas in P0528T, biotinylated proteins were significantly enhanced after labeling. The labeling effect was comparable at room temperature (28°C) and 37°C (Figures 3a,b). These results indicate that the in-frame expression of TurboID downstream of Sll0528 functioned as a biotin ligase *in vivo*, and the room temperature was selected as the convenient labeling temperature. When biotin concentration increased from 1 μM to 700 μM, the quantity of biotinylated proteins increased sharply from 1 μM to 100 μM, followed by a gradual leveling off from 100 μM to 700 μM (Figure 3b and Supplementary Figures S3c,d). Therefore, to minimize false negatives and false positives in the search for proximate proteins, 500 μM biotin was used as the labeling substrate. A comparison of labeling times from 6 min to 4 h suggested that TurboID performed biotinylation rapidly, as longer labeling periods did not significantly enhance the labeling effect (Supplementary Figure S3e). Thus, 15 min was chosen as the labeling duration for convenience and consistency.

The enrichment of biotinylated proteins was optimized in P0528T and control lines, TBYPC (Figure 3c and Supplementary Figure S3f). A desalting column was used to eliminate excess biotin, while a streptavidin affinity column was used to purify the biotinylated proteins. Un-biotinylated proteins were washed out through the column, while biotinylated proteins remained tightly bound. These proteins were finally eluted at high temperatures. The purified biotinylated proteins in P0528T showed sharp and clear patterns, ranging from 10 kD to more than 100 kD, with distinct bands present exclusively in P0528T and not in TBYPC. These findings suggest that the bands may be identified as Sll0528 proximate proteins in the proteomic analysis. TBYPC exhibited a similar biotinylated pattern to WT, indicating it could serve as a reliable background control for the subsequent experiments. There was no apparent pattern difference in the purified biotinylated proteins from P0528T, regardless of ammonium stress. Therefore, the question remains whether Sll0528 changes its interacting proteins during the early acclimation to ammonium stress.

### Identification of biotinylated proteins enriched in P0528T through proteomic analysis

**Figure 4:**
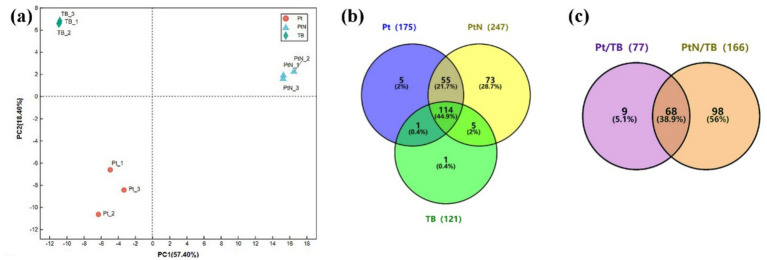
Identification of biotinylated proteins from P0528T and TBYPC lines. **(a)** PCA analysis of samples from three groups: Pt, PtN, and TB. Pt or PtN was P0528T without or with 30 min treatment of 90 mM NH_4_Cl. TB combined samples from TBYPC with and without ammonium stress as a background control. **(b)** Distribution of identified proteins among three groups. Protein detected in any of the three replicates of each group was defined as existing; otherwise, it was absent in this group. **(c)** Significantly enriched proteins in P0528T compared with TBYPC. Proteins exclusively detected in Pt and PtN, but not in TB, and proteins significantly accumulated in Pt or PtN compared with TB (FC ≥ 2, *p* ≤ 0.05), were included as significantly enriched proteins.

In the following proteomic analysis, approximately 2.2 mg to 4.3 mg of biotinylated proteins purified from TBYPC (TB), P0528T without ammonium stress (Pt), and P0528T with ammonium stress (PtN) were subjected to 4D label-free proteomics (Supplementary Table S3). The TB samples included proteins from TBYPC both without and with ammonium stress, serving as a background control. A total of 254 proteins were detected across the three groups (Figure 4 and Supplementary Table S4). PCA analysis of replicates from these groups showed that replicates within each group clustered together, while the different groups were clearly separated, indicating good reproducibility within groups and significant differences among proteins from TB, Pt, and PtN. The distribution of identified proteins among the three groups revealed that about half could be found in the TB group, which may include the background biotinylated proteins, while the other half (52.4%) were detected exclusively in Pt and/or PtN (Figure 4b). Only one protein found in the TB group was absent in both Pt and PtN, suggesting a low risk of false negatives. Interestingly, 21.7% of the proteins were detected in both Pt and PtN, while a larger portion (28.7%) was detected exclusively in PtN, significantly more than the 2% found exclusively in the Pt group. This could be due to the ammonium-induced proximate proteins around Sll0528, although it cannot be overlooked that the enhanced expression of Sll0528 under ammonium stress may have increased the biotin labeling radius to encompass more distant proteins. Consistent with our anticipation, Sll0528 was detected at high abundance in both Pt and PtN groups but was absent in TB, confirming that the TurboID expressed downstream of Sll0528 functioned as a biotin ligase to biotinylate Sll0528 itself and its proximate proteins, thus indicating that the proteomic data obtained were reliable and promising.

Other than the exclusively detected proteins in the P0528T line, some proteins were significantly enriched in PT/TB or PtN/TB (FC ≥ 2, *p* ≤ 0.05). As a result, these were included as enriched proteins for further verification (Figure 4c). Based on their EggNOG functional categories, with the exception of the largest category labeled as function unknown, the enriched proteins were primarily associated with energy production and conversion, ribosomal structure and biogenesis, as well as amino acid and nucleotide transport and metabolism. Additionally, it is noteworthy that a few dozen proteins were involved in posttranslational modification, chaperones, and signal transduction mechanisms (Supplementary Figure S4a). In contrast to the enriched biotinylated proteins in Pt and/or PtN, the functional classification of the remaining background biotinylated proteins (Supplementary Figure S4b) indicated their primary involvement in energy production and conversion, ribosomal biogenesis, and carbohydrate metabolism. This could be attributed to their high abundances within the cell, such as the Rubisco large and small subunits (Slr0009 and Slr0012) and numerous ribosomal proteins. Alternatively, it may be due to their natural affinity for biotin, as observed with Slr0435 (the biotin carboxyl carrier protein of acetyl-CoA carboxylase) and Sll1841 (the dihydrolipoamide acetyltransferase component of the pyruvate dehydrogenase complex), both of which are enzymes utilizing biotin as a cofactor.

**Figure 5:**
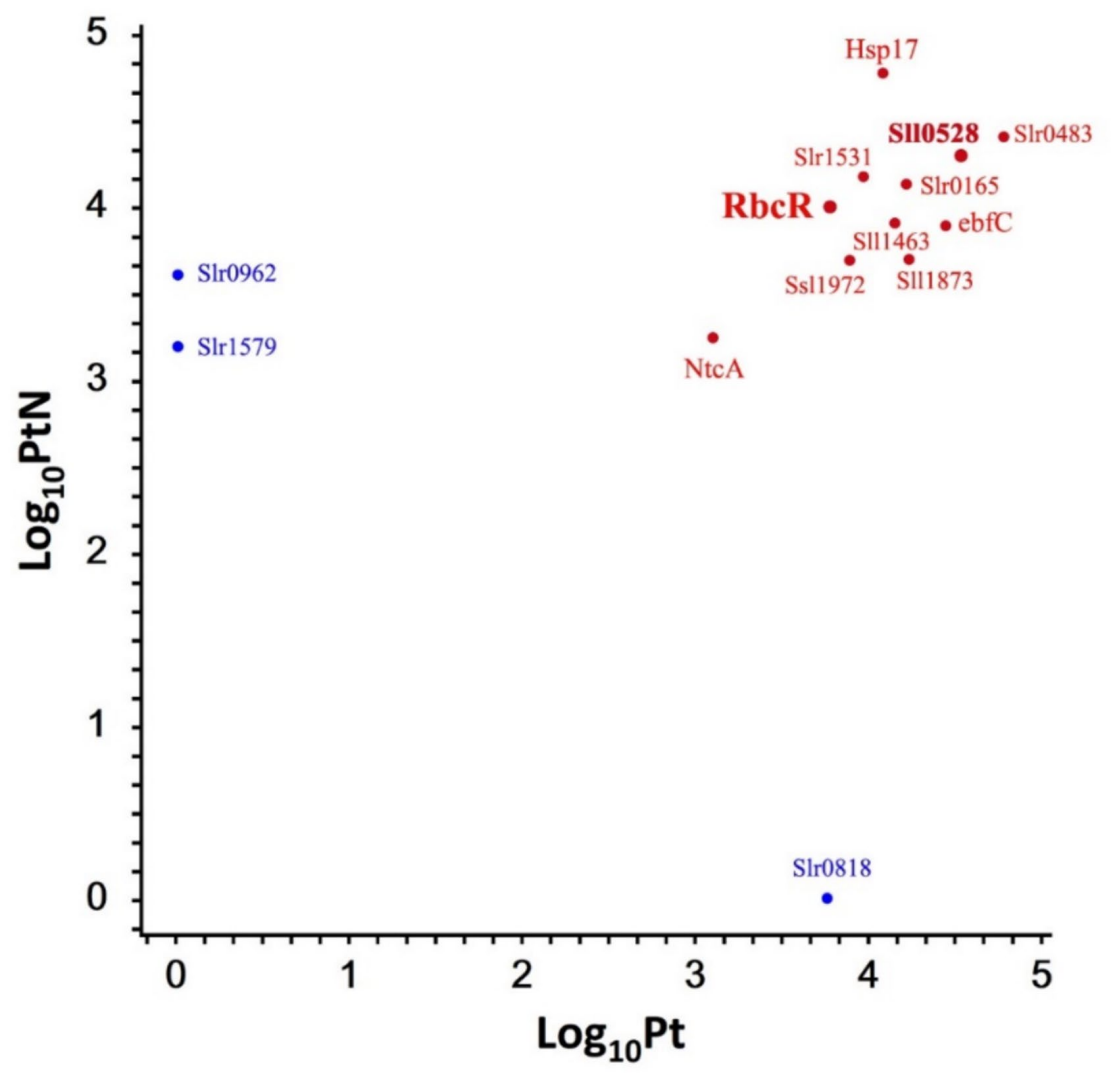
Representative proteins enriched in P0528T without or with ammonium stress. A scatterplot of the log_10_ protein amount of representative proteins was detected in P0528T without (Pt) or with ammonium stress (PtN). Enriched proteins exclusively detected in Pt or PtN but not in TB are indicated in blue; otherwise, they are indicated in red.

A total of 175 proteins were enriched in Pt and/or PtN (Supplementary Figure S5). To further verify the Sll0528 interacting protein, we selected the enriched proteins involved in posttranslational modification, chaperones, and signal transduction mechanisms, as well as those assigned as unknown, for further verification (Figure 5).

### Bacterial two-hybrid assay

**Figure 6:**
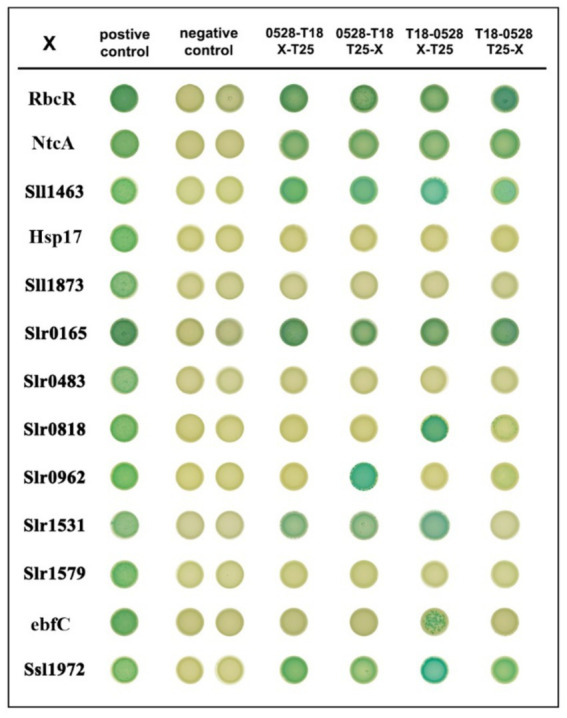
Bacterial two-hybrid interactions on X-Gal plates of Sll0528 with different proximate proteins. The interaction of leucine zippers of GCN4 from the plasmids pKT25-zip and pUT18C-zip was used as a positive control. Dimerization of the leucine zipper motifs appended to the T25 and T18 fragment results in the positive association of T25 and T18 (1st column). Positive interactions are indicated as blue colonies. Interaction of pUT18C-sll0528 with an empty pKT25 vector was used as a negative control in the 2nd (T18 appended to the N terminal of Sll0528) and 3rd (T18 appended to the C terminal of Sll0528) columns. Different combinations of plasmids included T18 appended to the C terminal of Sll0528 (4th and 5th columns) or T18 appended to the N terminal of Sll0528 (6th and 7th columns) co-expressed with T25 appended to the N terminal (4th and 6th columns) or C terminal (5th and 7th columns) of different proximate proteins (X).

To validate the interaction of Sll0528 with the identified proximate proteins, a bacterial two-hybrid assay (BACTH) was utilized based on adenylate cyclase reconstitution from T18 and T25 fragments in *E. coli* (Battesti and Bouveret, 2012). To avoid interference with the fusion tag, the T18 fragment was fused with Sll0528 at either its N-or C-terminal. A total of 13 proximate proteins (Figure 5) were selected to test for possible interactions, with the T25 fragment fused at either their N-or C-terminal. Dimerization of the leucine zipper served as a positive control, while the negative control involved Sll0528 fused with T-18 and co-expressed with an empty pKT25. Nine out of the 13 proximate proteins demonstrated positive interaction on X-Gal plates in at least one of the four BACTH combinations, including the transcription factor RbcR (Sll0998) (Figure 6). Negative results were observed in BACTH for the unknown proteins Slr1579, Slr0483, Sll1873, and the heat shock protein, molecular chaperone Hsp17 (Sll1514). These may be adventitious proteins associated with Sll0528, or they may not have been properly validated for interaction with Sll0528 in BACTH. For instance, the immunoprecipitation pull-down protein UrtD showed negative results in BACTH (Watzer et al., 2019). Other than RbcR, positive interactions in BACTH included the unknown proteins Ssl1972, Slr0962, Slr0818, the nucleoid-associated protein ebfC (Slr1847), signal recognition particle protein Slr1531, the probable Clp protease subunit Slr0165, zinc metalloprotease FtsH (Sll1463), and the global nitrogen regulator NtcA (Sll1423).

### Transcriptomics demonstrates RbcR regulon downregulated in *sll0528* knockout mutant

The transcriptomic analysis of the *sll0528* knockout mutant *Δsll0528* and overexpression strain *OEsll0528* was then conducted to compare with WT. Significantly lower or higher *sll0528* mRNA abundance in *Δsll0528* or *OEsll0528,* respectively, confirmed the mutant construction as expected (Table 1). Knockout or overexpression of the *sll0528* gene did not significantly change the transcript level of the 13 proximate proteins analyzed in BACTH, including RbcR (Table 1 and Supplementary Table S5). However, *rbcLXS* encoding Rubisco subunit and its chaperonin and *sll0519* encoding NADH dehydrogenase subunits had diminished transcript abundance in *Δsll0528* compared to WT but not in *OEsll0528* compared to WT (Table 1). Since RuBisCO genes were controlled by RbcR and were part of the RbcR regulon (Bolay et al., 2022), we further analyzed the other RbcR regulon genes (Table 1). Intriguingly, the group of RbcR regulon genes that were significantly downregulated in the partially segregated *rbcR* knockout mutant were also dramatically downregulated in *Δsll0528*. This includes genes from the *ccmK2K1LMN* operon that encode carboxysome elements, the *slr1279/80/81* genes that encode NADH dehydrogenase subunits, and *sbtA,* which encodes the high-affinity bicarbonate transporter.

**Table 1:** Transcriptomic level of *sll0528*, *rbcR,* and the RbcR regulon in the *Δsll0528* and *OEsll0528* compared to wild type.

Gene ID	Gene name	Annotation	Log_2_FoldChange M vs. WT	Log_2_FoldChange OE vs. WT
*sll0528*		S2P, site-2 protease	−1.70	3.64
*sll0998*	*rbcR*	RuBisCO transcriptional regulator RbcR	−0.06	−0.20
*sll0519*	*ndhA*	NADH dehydrogenase subunit 1	−1.37	−0.46
*sll1028*	*ccmK*	Carbon dioxide-concentrating mechanism protein CcmK	−0.72	−0.08
*sll1029*	*ccmK*	Carbon dioxide-concentrating mechanism protein CcmK	−0.76	−0.13
*sll1030*	*ccmL*	Carbon dioxide-concentrating mechanism protein CcmL, putative carboxysome assembly protein	−0.72	−0.13
*sll1031*	*ccmM*	Carbon dioxide-concentrating mechanism protein CcmM, putative carboxysome structural protein	−0.88	−0.22
*sll1032*	*ccmN*	Carbon dioxide-concentrating mechanism protein CcmN, putative carboxysome assembly protein	−0.93	−0.32
*sll1735*		Secreted protein MPB70	−2.27	−1.77
*slr0006*		Unknown protein	−2.29	−1.50
*slr0007*		Probable sugar-phosphate nucleotidyltransferase	−2.22	−1.62
*slr0009*	*rbcL*	RuBisCO large subunit	−1.26	−0.26
*slr0011*	*rbcX*	possible RuBisCO chaperonin	−1.15	−0.23
*slr0012*	*rbcS*	RuBisCO small subunit	−0.99	−0.13
*slr0476*		Unknown protein	−1.02	−0.42
*slr1279*	*ndhC*	NADH dehydrogenase subunit 3	−1.47	−0.71
*slr1280*	*ndhK*	NADH dehydrogenase subunit NdhK	−1.52	−0.88
*slr1281*	*ndhJ*	NADH dehydrogenase subunit I	−1.71	−0.93
*slr1512*	*sbtA*	Sodium-dependent bicarbonate transporter	−2.66	−0.84
*slr1513*	*sbtB*	PII-like signaling protein	−2.17	−0.64

M, Δsll0528. OE, OEsll0528. WT, wild type.

RuBisCO, ribulose-1,5-bisphosphate-carboxylase/oxygenase.

Meanwhile, the downregulation vanished or diminished in *OEsll0528*. So far, RbcR was the only transcriptional regulator known to control RuBisCO and was indispensable for the viability of *Synechocystis* (Bolay et al., 2022). Unlike the lowered *rbcR* mRNA abundance in the partially segregated *rbcR* knockout mutant in the previous report (Bolay et al., 2022), similar *rbcR* mRNA abundance was observed here in *Δsll0528* and WT. Thus, the compromised RbcR regulon expression might be due to the improper post-transcriptional regulation of RbcR, probably related to its interaction with Sll0528, as suggested by the proximity labeling and BACTH (Figures 5, 6). It is tempting to propose that the full functionality of RbcR to activate its regulon might depend on the functional Sll0528. Thus, the sll0528 knockout resulted in compromised RbcR regulon expression.

## Discussion

In this study, TurboID-based proximity labeling was applied, revealing a robust set of putative interacting proteins of Sll0528 for functional characterization. Among the interactions verified by BACTH, the transcriptional regulator RbcR, which controls the transcription of the *rbcLXS* operon, was identified. The interaction between Sll0528 and RbcR was further supported by the transcriptional analysis of the RbcR regulon. Interestingly, the transcriptomic data indicated that the RbcR regulon was downregulated in the *sll0528* knockout mutant, while its expression was not upregulated in the *sll0528* overexpression strain. This observation was consistent with the previous report of the *rbcR* overexpression strain in which only the *rbcR* gene itself and transcripts associated with this locus were differentially expressed (Bolay et al., 2022). This might result from the saturation of all potential DNA binding sites with RbcR, as the overexpression of RbcR (Bolay et al., 2022) or Sll0528 here has little effect on the transcriptional landscape.

RbcR positively controlled the RuBisCO encoding genes, as well as several other genes encoding components of C_i_ assimilation and concentration in *Synechocystis*; thus, RbcR was recognized as the third major regulator of cyanobacterial C_i_ assimilation, in addition to transcription factor NdhR and CmpR (Bolay et al., 2022). NdhR (also called CcmR) is a carbon-responsive repressor that acts on the bicarbonate transporter operon in high CO_2_ conditions (Wang et al., 2004). NdhR is activated by 2-oxoglutarate (2-OG) that accumulates in cells when the Ci/nitrogen ratio is high, while it is inhibited by 2-phosphoglycolate (2-PG), the first intermediate of the photorespiration, indicating low Ci (Jiang et al., 2018). RbcR, CmpR, and cyAbrB2 activate transcription of the bicarbonate transporter operon under low-carbon conditions (Nishimura et al., 2008; Orf et al., 2016; Bolay et al., 2022). For the known carbon-responding transcriptional regulator, RbcR is the main regulator for *rbcLXS* and *ccmK2K1LMN* operons in low Ci, whereas the *rbcLXS* and *ccmK2K1LMN* operons do not belong to the NdhR, CmpR or CyAbrB2 regulons (Bolay et al., 2022). A high concentration of ammonium actually results in a relatively low intracellular C_i_ supply. Interestingly, among the four Ci assimilation-related transcriptional regulators, only RbcR was significantly induced upon 30 mM NH_4_Cl (Lin et al., 2023), consistent with its active engagement in ammonium acclimation. RbcR has homologs widely distributed among cyanobacteria, such as PacR in the filamentous, diazotrophic cyanobacterium *Anabaena* sp. PCC 7120.

PacR was first discovered as a global regulator of photosynthetic carbon assimilation in *Anabaena* (Picossi et al., 2015) and was recently reported to control heterocyst differentiation, serving as a crucial transcriptional regulator for balancing carbon and nitrogen metabolism (Lin et al., 2025). Intriguingly, search for the Sll0528 homolog in *Anabaena* sp. PCC 7120 revealed at least two candidates, All1844 and Alr3900, both of which contain the conserved HELGH motif within one of the transmembrane domains. It is tempting to propose that the interaction between Sll0528 and RbcR, observed in *Synechocystis,* might be conserved among cyanobacteria and play a role in regulating carbon and nitrogen metabolism. In this context, RbcR was identified as the Sll0528-interacting protein, which provides novel insight for investigating carbon/nitrogen homeostasis control during ammonium stress acclimation. However, the role of the interaction between Sll0528 and RbcR in carbon/nitrogen homeostasis control under ammonium stress remains to be further explored. Meanwhile, it cannot be ruled out that Sll0528 may interact with additional transcriptional regulators to control this group of genes, including *rbcLXS*. If this is the case, further investigation of other Sll0528 proximity proteins could uncover a novel regulator of *rbcLXS*.

NtcA was identified as the proximity protein of Sll0528, and its interaction with Sll0528 was confirmed using BACTH (Figures 5, 6). NtcA is the global transcriptional regulator that controls nitrogen assimilation and metabolism under nitrogen depletion (Vega-Palas et al., 1992; García-Domínguez et al., 2000; Forcada-Nadal et al., 2018). Fifty-one genes activated and 28 repressed directly by NtcA during early acclimation to nitrogen starvation were identified through a combination of chromatin immunoprecipitation sequencing and transcriptomic analysis (Giner-Lamia et al., 2017). However, since the acclimation to ammonium stress is not merely the reverse of nitrogen starvation (Lin et al., 2023), given the data in hand, the involvement of NtcA in ammonium acclimation through its interaction with Sll0528 remains obscure and is awaiting further investigation.

Our study here establishes the site-2 protease Sll0528 as an essential regulator of ammonium stress acclimation in *Synechocystis* sp. PCC 6803. Proximity labeling of Sll0528 revealed a robust set of putative Sll0528-interacting proteins for functional characterization. Some of the interactions were subsequently confirmed through the bacterial two-hybrid assay, including the transcriptional regulator RbcR. Transcriptomic analysis indicated the significant downregulation of the RbcR regulon in the *sll0528* knockout mutant, suggesting that the compromised RbcR regulon expression was due to the improper post-transcriptional regulation of RbcR, mediated through the interaction with Sll0528. They cooperate to regulate carbon/nitrogen homeostasis and contribute to stress acclimation under ammonium, highlighting an important function of Sll0528 in metabolic control. The evolutionary conservation of S2P proteases across all domains of life underscores their fundamental regulatory importance. While cyanobacteria and plant S2Ps [e.g., *Arabidopsis* EGY1/AMOS1 regulating chloroplast development and nutrient stress response (Chen et al., 2005; Guo et al., 2008; Li et al., 2012; Yu et al., 2016), and tomato Lutescent2 controlling fruit ripening (Barry et al., 2012; Li et al., 2021)] demonstrate functional diversity, progress in understanding their mechanisms has been limited by unidentified downstream targets. Our findings reveal an interaction between a cyanobacterial S2P protease and the carbon-responsive transcriptional machinery, providing new mechanistic insights into the regulation of carbon-nitrogen homeostasis in cyanobacteria during nitrogen fluctuations. This provides hints for the functional characterization of other S2P proteases in photosynthetic organisms and may facilitate the cyanobacteria-based bioremediation of ammonium-rich wastewater in the future.

## Data Availability

The original datasets presented in the study are publicly available. The mass spectrometry proteomics data have been deposited to the ProteomeXchange Consortium via the PRIDE partner repository with the dataset identifier PXD062293.

## Footnotes

^1^ http://bowtie-bio.sourceforge.net/bowtie2/index.shtml

^2^ http://deweylab.github.io/RSEM/

^3^ http://www.bioconductor.org/packages/2.12/bioc/html/edgeR.html
