# Supplementary material for: Site-2 protease Sll0528 interacts with RbcR to regulate carbon/nitrogen homeostasis in the cyanobacterium Synechocystis sp. PCC 6803
Source: Front Microbiol. 2025 Apr 9;16:1556583. doi: 10.3389/fmicb.2025.1556583 (PMC12014562; doi:10.3389/fmicb.2025.1556583)
Supplement: Supplementary file 1 [file Data_Sheet_1.DOCX]

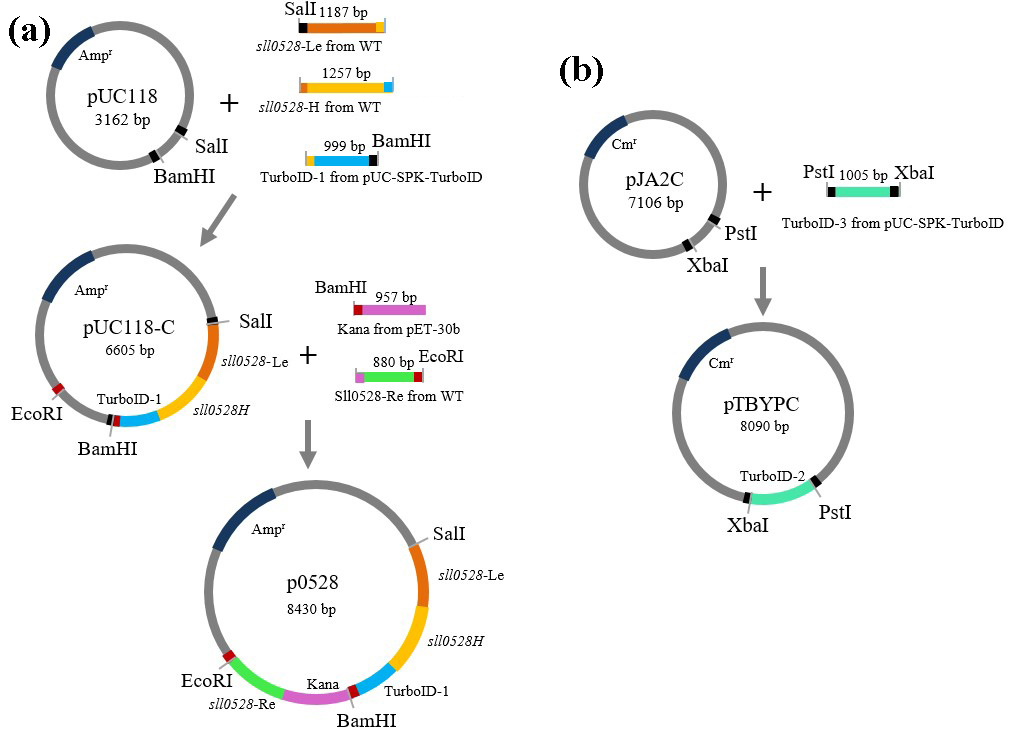


**Fig. S1** Construction of plasmids to generate P0528T and TBYPC line. (a) Construction of the plasmid p0528 for preparing P0528T line. The TurboID gene was fused in frame with *sll0528* gene under the *sll0528* promoter. (b) Construction of the plasmid pTBYPC for preparing TBYPC line. The TurboID gene was expressed under the psbA2 promoter.


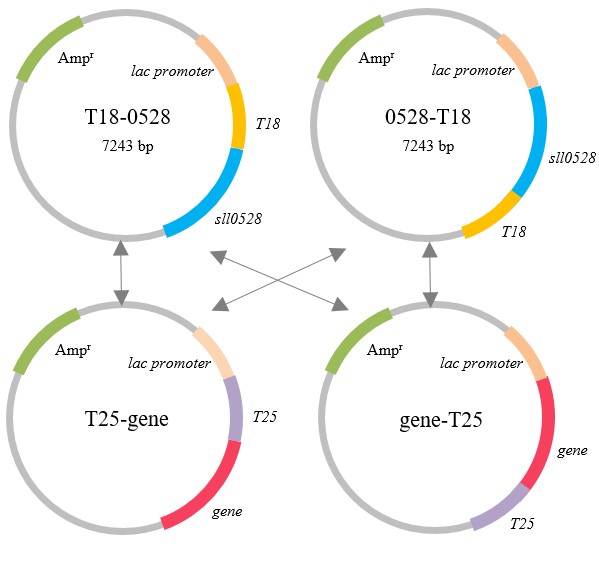


**Fig. S2** Plasmid construction and co-transformation for the bacterial two-hybrid assay. T18 and T25 are components of the adenylate cyclase (CyaA) enzyme. The gene of interest (POI) is fused to either the N- or C-terminal end of either T18 or T25 subunit. The CyaA enzyme is inactive when T18 and T25 are physically separated. However, when the POIs interact, the proximity of T18 and T25 allows for the production of cyclic adenosine monophosphate (cAMP). This cAMP then binds to catabolic activation protein (CAP), resulting cAMP/CAP complex activates the transcription of reporter genes to be detected. The gray arrows in the figure indicate the combination of co-transformation.

**
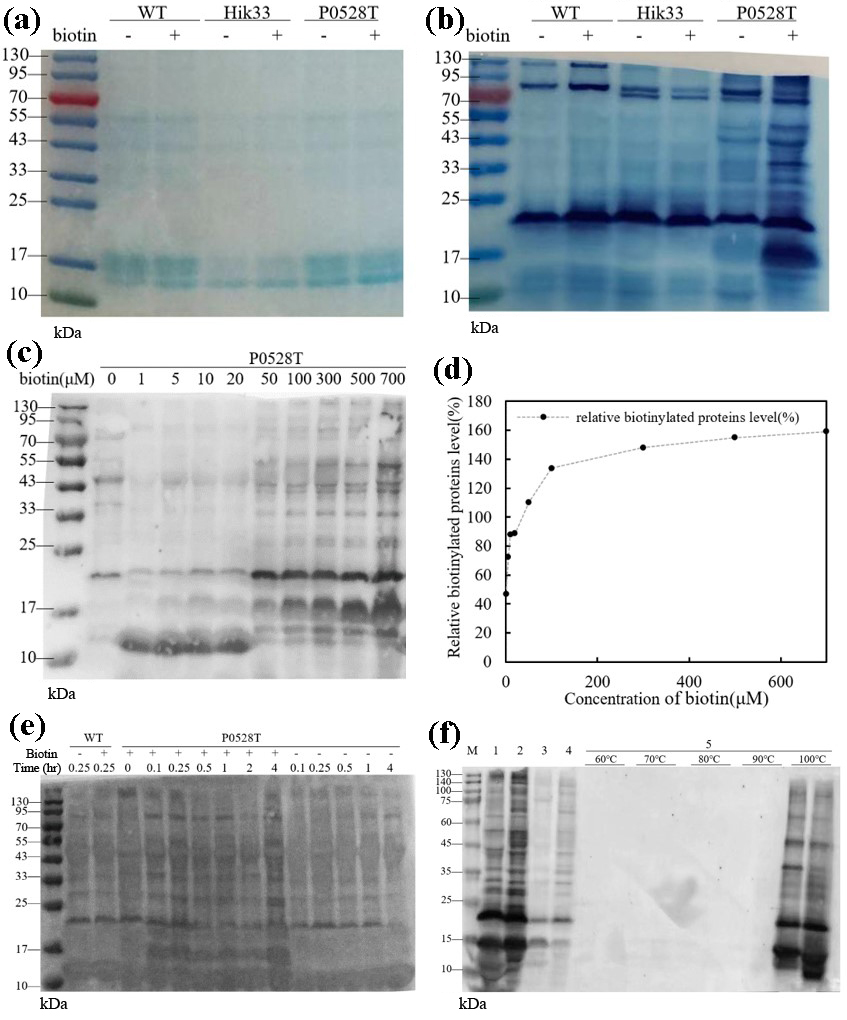
**

**Fig. S3** Optimization of biotin labeling and enrichment of biotinylated proteins. Biotinylated proteins were separated in SDS-PAGE and revealed by antibody Streptavidin-HRP in western blot. (a) SDS-PAGE of total protein extracted from WT (wild type), Hik33 line (a control line that TurboID expressed downstream of soluble protein Hik33), and P0528T line without (-) or with (+) biotin labeling. (b) Western blot of total protein extracted from WT, Hik33 and P0528T without (-) or with (+) biotin labeling. (c) Comparison of labeling effects with different concentration of biotin under RT in P0528T. (d) Quantification of the relative level of biotinylated proteins in P0528T from C. (e) Comparison of biotin labeling effect with different labeling time in WT and P0528T. (f) Purification of biotinylated proteins through the streptavidin affinity column. 1 to 5 indicated sample during purification steps. 1: protein sample before desalting column; 2: protein sample after desalting column; 3: protein sample before streptavidin affinity column; 4: protein sample after streptavidin affinity column; 5: eluate after in-column incubation. Before step 5, the streptavidin affinity column with elution buffer was incubated at specific temperature and their effect on final eluate was compared.


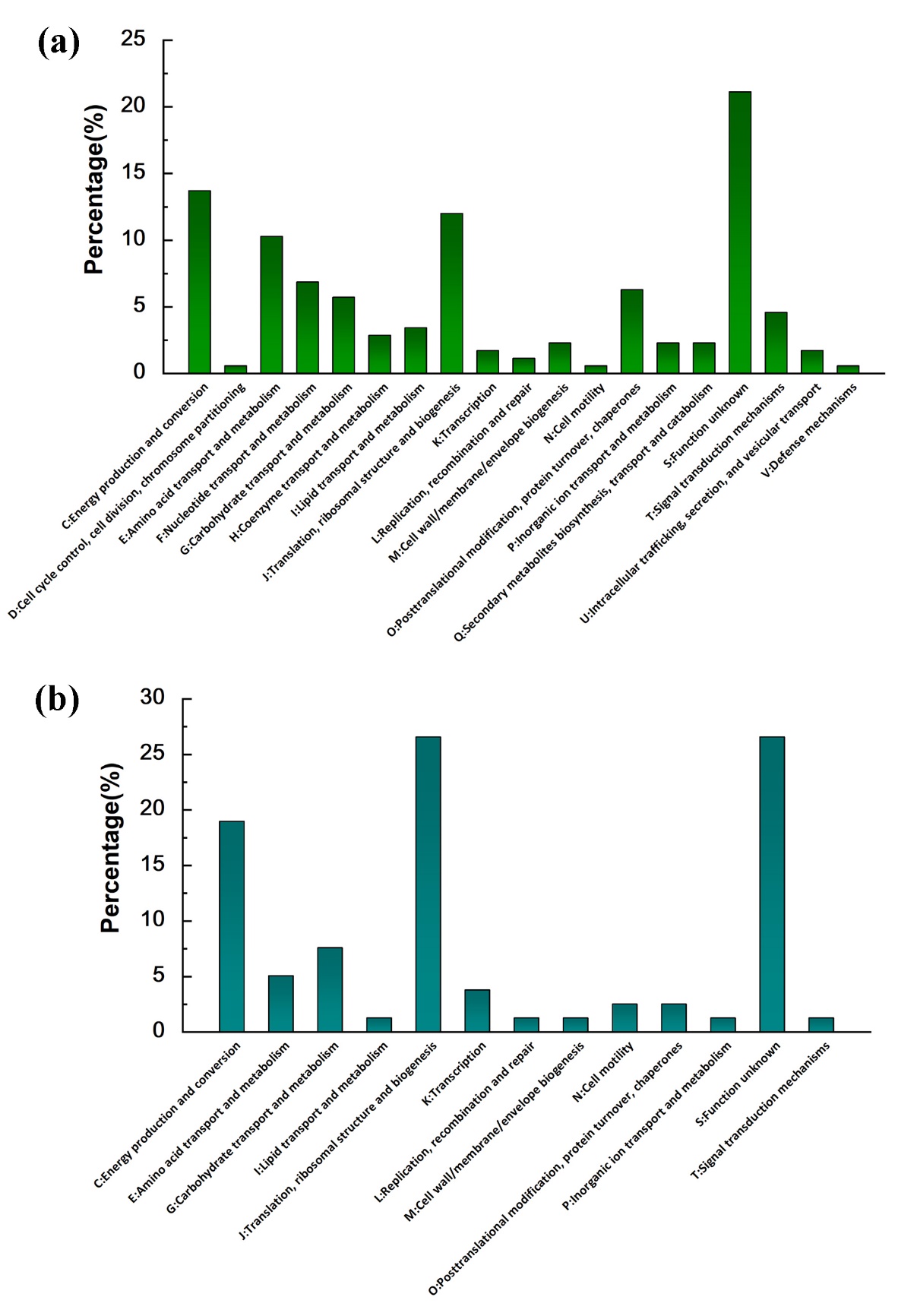


**Fig. S4** Functional Classification of biotinylated proteins. (a) Functional Classification of 175 enriched proteins in P0528T line as indicated in Fig.4C. (b) Functional Classification of the background biotinylated proteins.

**
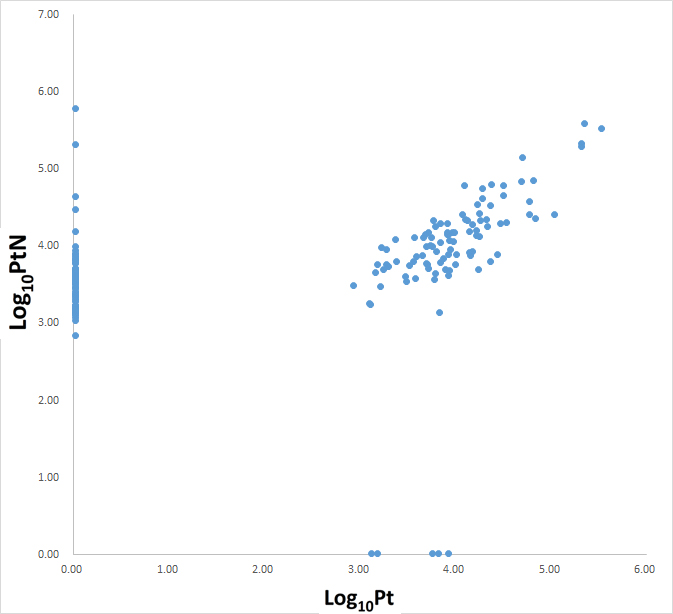
**

**Fig. S5.** Proteins enriched in P0528T without or with ammonium stress. Scatterplot of the log_10_ protein amount of enriched proteins detected in P0528T without (Pt) or with ammonium stress (PtN).

**Table S1** Plasmids used in this study.

| **Plasmid** | **Tag localization** | **Description** |
| --- | --- | --- |
| p0528 |  | For generation of recombinant *Synechocystis* strain P0528T |
| pTBYPC |  | For generation of recombinant *Synechocystis* strain TBYPC |
| pUC-SPK-TurboID | | Contain the TurboID gene generated by DNA synthesis |
| pUT18 |  | Encoding T18 fragment of adenylate cyclase CyaA |
| pKT25 |  | Encoding T25 fragment of adenylate cyclase CyaA. Negative control |
| pUT18-zip | N-terminal | Derived from pUT18. Sequence coding for the leucine zipper region of the GCN4 yeast protein. Positive control |
| pKT25-zip | N-terminal | Derived from pKT25. Sequence coding for the leucine zipper region of the GCN4 yeast protein. Positive control |
| Sll0528-T18 | C-terminal | Derived from pUT18. Sequence coding for protein Sll0528 |
| T18-Sll0528 | N-terminal | Derived from pUT18. Sequence coding for protein Sll0528 |
| Sll1423-T25 | C-terminal | Derived from pKT25. Sequence coding for protein Sll1423 |
| T25-Sll1423 | N-terminal | Derived from pKT25. Sequence coding for protein Sll1423 |
| Sll0998-T25 | C-terminal | Derived from pKT25. Sequence coding for protein Sll0998 |
| T25-Sll0998 | N-terminal | Derived from pKT25. Sequence coding for protein Sll0998 |
| Sll1463-T25 | C-terminal | Derived from pKT25. Sequence coding for protein Sll1463 |
| T25-Sll1463 | N-terminal | Derived from pKT25. Sequence coding for protein Sll1463 |
| Sll1514-T25 | C-terminal | Derived from pKT25. Sequence coding for protein Sll1514 |
| T25-Sll1514 | N-terminal | Derived from pKT25. Sequence coding for protein Sll1514 |
| Sll1873-T25 | C-terminal | Derived from pKT25. Sequence coding for protein Sll1873 |
| T25-Sll1873 | N-terminal | Derived from pKT25. Sequence coding for protein Sll1873 |
| Slr0165-T25 | C-terminal | Derived from pKT25. Sequence coding for protein Slr0165 |
| T25-Slr0165 | N-terminal | Derived from pKT25. Sequence coding for protein Slr0165 |
| Slr0483-T25 | C-terminal | Derived from pKT25. Sequence coding for protein Slr0483 |
| T25-Slr0483 | N-terminal | Derived from pKT25. Sequence coding for protein Slr0483 |
| Slr0818-T25 | C-terminal | Derived from pKT25. Sequence coding for protein Slr0818 |
| T25-Slr0818 | N-terminal | Derived from pKT25. Sequence coding for protein Slr0818 |
| Slr0962-T25 | C-terminal | Derived from pKT25. Sequence coding for protein Slr0962 |
| T25-Slr0962 | N-terminal | Derived from pKT25. Sequence coding for protein Slr0962 |
| Slr1531-T25 | C-terminal | Derived from pKT25. Sequence coding for protein Slr1531 |
| T25-Slr1531 | N-terminal | Derived from pKT25. Sequence coding for protein Slr1531 |
| Slr1579-T25 | C-terminal | Derived from pKT25. Sequence coding for protein Slr1579 |
| T25-Slr1579 | N-terminal | Derived from pKT25. Sequence coding for protein Slr1579 |
| Slr1847-T25 | C-terminal | Derived from pKT25. Sequence coding for protein Slr1847 |
| T25-Slr1847 | N-terminal | Derived from pKT25. Sequence coding for protein Slr1847 |
| Ssl1972-T25 | C-terminal | Derived from pKT25. Sequence coding for protein Ssl1972 |
| T25-Ssl1972 | N-terminal | Derived from pKT25. Sequence coding for protein Ssl1972 |

**Table S2** Sequence of primers used in cloning and RT-qPCR.

| **Name** | **Primer（5’-3’）** |
| --- | --- |
| sll0528-Le-F | CTTGCATGCCTGCAGGTCGACCTGGTTATGGTGGTTTACTGAG |
| sll0528-Le-R | ATGATGATGATGCATGAATTAATTGTAACGAGATGTTAAG |
| sll0528-H-F | CGTTACAATTAATTCATGCATCATCATCATCATCATCATC |
| sll0528-H-R | GGTGTTATCCTTCATGGAGCCGCCGCCGCCGGAG |
| sll0528-Re-F | GATGAGTTTTTCTAAAGCCTGTTTTAAAAGCCC |
| sll0528-Re-R | TATGACCATGATTACGAATTCGTGGAAATGGCCAAAACTACTC |
| TurboID-F | TACATAAGGAATTATTCTAGAATGAAGGATAACACCGTGC |
| TurboID-R | TTATTTGATGCCTGGCTGCAGTTATTTTTCTGCACTACGCA |
| Kana-F | GATGAGTTTTTCTAAAGCCTGTTTTAAAAGCCC |
| Kana-R | TATGACCATGATTACGAATTCGTGGAAATGGCCAAAACTACTC |
| 0528-T18-IN-F | ATTTCACACAGGAAACAGCTATGTTAAGCCTCAGTTTAGGGGG |
| 0528-T18-IN-R | CTTGGCGTAATCATGGTCATGGCGGCGGAGGTTTGCAG |
| 0528-T18-VE-F | ATGACCATGATTACGCCAAGC |
| 0528-T18-VE-R | AGCTGTTTCCTGTGTGAAATTGTT |
| T18-0528-IN-F | CAGTGGAACGCCAATCGATAATGTTAAGCCTCAGTTTAGGGGG |
| T18-0528-IN-R | AGCGACCGGCGCTCAGCTTACTAGGCGGCGGAGGTTTG |
| T18-0528-VE-F | TAAGCTGAGCGCCGGTCG |
| T18-0528-VE-R | TATCGATTGGCGTTCCACTGC |
| gene-T18-VET | ATGACCATGCAGCAATCGCA |
| gene-T18-VET | AGCTGTTTCCTGTGTGAAATTGTT |
| T18-gene-VET | TGACTGGGAAAACCCTGGC |
| T18-gene-VET | CGACGTTGTAAAACGACGGC |
| 0998-T25-IN-F | ATTTCACACAGGAAACAGCTATGTCGGATATCCCGTTCACG |
| 0998-T25-IN-R | TGCGATTGCTGCATGGTCATAGTAACCGAGTCGATTTCAATGC |
| T25-0998-IN-F | GCCGTCGTTTTACAACGTCGATGTCGGATATCCCGTTCACG |
| T25-0998-IN-R | CGCCAGGGTTTTCCCAGTCAAGTAACCGAGTCGATTTCAATGC |
| 1423-T25-IN-F | ATTTCACACAGGAAACAGCTATGGATCAGTCCCTAACCCAAGA |
| 1423-T25-IN-R | TGCGATTGCTGCATGGTCATGGTAAACTGTTGACTGAGAGCAACG |
| T25-1423-IN-F | GCCGTCGTTTTACAACGTCGATGGATCAGTCCCTAACCCAAGA |
| T25-1423-IN-R | CGCCAGGGTTTTCCCAGTCAGGTAAACTGTTGACTGAGAGCAACG |
| 1463-T25-IN-F | ATTTCACACAGGAAACAGCTATGGCCATCAAACCCCAAC |
| 1463-T25-IN-R | TGCGATTGCTGCATGGTCATTACCACTAGGGTGCCAGGAGC |
| T25-1463-IN-F | GCCGTCGTTTTACAACGTCGATGGCCATCAAACCCCAAC |
| T25-1463-IN-R | CGCCAGGGTTTTCCCAGTCATACCACTAGGGTGCCAGGAGC |
| 0165-T25-IN-F | ATTTCACACAGGAAACAGCTATGCCTATAGGTGTTCCCAGTGTT |
| 0165-T25-IN-R | TGCGATTGCTGCATGGTCATGTTATTGCCCATGGTGGAGTTT |
| T25-0165-IN-F | GCCGTCGTTTTACAACGTCGATGCCTATAGGTGTTCCCAGTGTT |
| T25-0165-IN-R | CGCCAGGGTTTTCCCAGTCAGTTATTGCCCATGGTGGAGTTT |
| 1972-T25-IN-F | ATTTCACACAGGAAACAGCTATGATCAGCATTGATTTGACCCT |
| 1972-T25-IN-R | TGCGATTGCTGCATGGTCATACCTTGATTAACAATTTGGCCC |
| T25-1972-IN-F | GCCGTCGTTTTACAACGTCGATGATCAGCATTGATTTGACCCT |
| T25-1972-IN-R | CGCCAGGGTTTTCCCAGTCAACCTTGATTAACAATTTGGCCC |
| 0528-T18-yzF | TGGTTCCTCCGCCCGCAA |
| 0528-T18-yzR | ACTTCCAGCTCGAAATCGGT |
| T18-0528-yzF | TTTCGTCGTATCGGCCACC |
| T18-0528-yzR | ACTCGCTAGACCACCGAAC |
| 0165-T25-check | TCAAGCAACGGACATTGACA |
| 0165-T25-check | CTTGCCGCAGATAGTCAAGC |
| T25-0165-check | TCTGTCCAACTTCCGCGACT |
| T25-0165-check | AATTTCCCTGGCTTCAATGTCA |
| 1972-T25-check | CACTTTATGCTTCCGGCTCGT |
| 1972-T25-check | GCAGATAGTCAAGCCGCTCT |
| T25-1972-check | CCACCAGCCTGATTGCCGAAG |
| T25-1972-check | CTCCGAAACAATGACCGCACT |
| 0998-T25-check | TTAGCCTCGATTCCCAGTCCAC |
| 0998-T25-check | GCAGATAGTCAAGCCGCTCT |
| T25-0998-check | TCTGTCCAACTTCCGCGACT |
| T25-0998-check | ACCGTTACGTCAGGATATTGCT |
| 1423-T25-check | ATGATTGAAACCCTAGCCCAC |
| 1423-T25-check | GCAGATAGTCAAGCCGCTCT |
| T25-1423-check | TCTGTCCAACTTCCGCGACT |
| T25-1423-check | GGCAGGTACACCAAAATCTCGAC |
| 1463-T25-check | ACACTGCCAAAGAAATTGACC |
| 1463-T25-check | GCAGATAGTCAAGCCGCTCT |
| T25-1463-check | ATCTGTCCAACTTCCGCGACT |
| T25-1463-check | CCACCACTTCGCTCAATTCCG |
| P0528T-S | CTCTTTGCTTCCGTTGTCGC |
| P0528T-A | CTTGCCAGGGAGTGTTGGAT |
| TBYPC-S | ACTGGGGCGTTGATGTCTTT |
| TBYPC-A | TCGCCCGATTTAAGCTCTCC |
| rnpB-S | GAGTTAGGGAGGGAGTTGCG |
| rnpB-A | GTGCAGGATGACGGAGAAAT |

**Table S3** Enriched biotinylated protein samples for proteomic analysis.

| **Sample ID** | **Protein concentration (μg/μL)** | **Protein amount (μg)** |
| --- | --- | --- |
| TB_1 | 7.575 | 3788 |
| TB_2 | 7.972 | 3986 |
| TB_3 | 8.769 | 4385 |
| Pt_1 | 5.756 | 2878 |
| Pt_2 | 6.002 | 3001 |
| Pt_3 | 6.628 | 3314 |
| PtN_1 | 4.404 | 2202 |
| PtN_2 | 7.814 | 3907 |
| PtN_3 | 6.053 | 3027 |

**Table S4** Identified biotinylated proteins. It was deposited in separate excel files.

**Table S5** Transcriptomic analysis of Sll0528 proximate protein gene.

| **Gene ID** | **Annotation** | **Log2FoldChange MvsWT** | **Log2FoldChange OEvsWT** |
| --- | --- | --- | --- |
| *sll1423* | global nitrogen regulator NtcA | 0.17 | 0.25 |
| *sll1463* | ATP-dependent zinc metalloprotease FtsH4 | -0.02 | 0.06 |
| *sll1514* | molecular chaperone heat shock protein | -0.28 | 0.95 |
| *sll1873* | unknown protein | 0.01 | 0.49 |
| *slr0165* | ATP-dependent Clp protease proteolytic subunit | -0.75 | -0.37 |
| *slr0483* | unknown protein | 0.03 | -0.29 |
| *slr0818* | unknown protein | -0.33 | -0.05 |
| *slr0962* | unknown protein | 0.40 | 0.13 |
| *slr1531* | signal recognition particle protein | 0.05 | 0.13 |
| *slr1579* | unknown protein | -0.10 | -0.03 |
| *slr1847* | nucleoid-associated protein | 0.08 | 0.27 |
| *ssl1972* | unknown protein | 0.12 | 0.09 |
